# Supplementary material for: Causal Relationship between Aging and Anorexia Nervosa: A White-Matter-Microstructure-Mediated Mendelian Randomization Analysis
Source: Biomedicines. 2024 Aug 16;12(8):1874. doi: 10.3390/biomedicines12081874 (PMC11351342; doi:10.3390/biomedicines12081874)
Supplement: Supplementary file 1 [file biomedicines-12-01874-s001.zip › Supplementary Figure/Figure S4/Singe SNP plot of MR analysis for TL to ubm-b-1519.pdf]

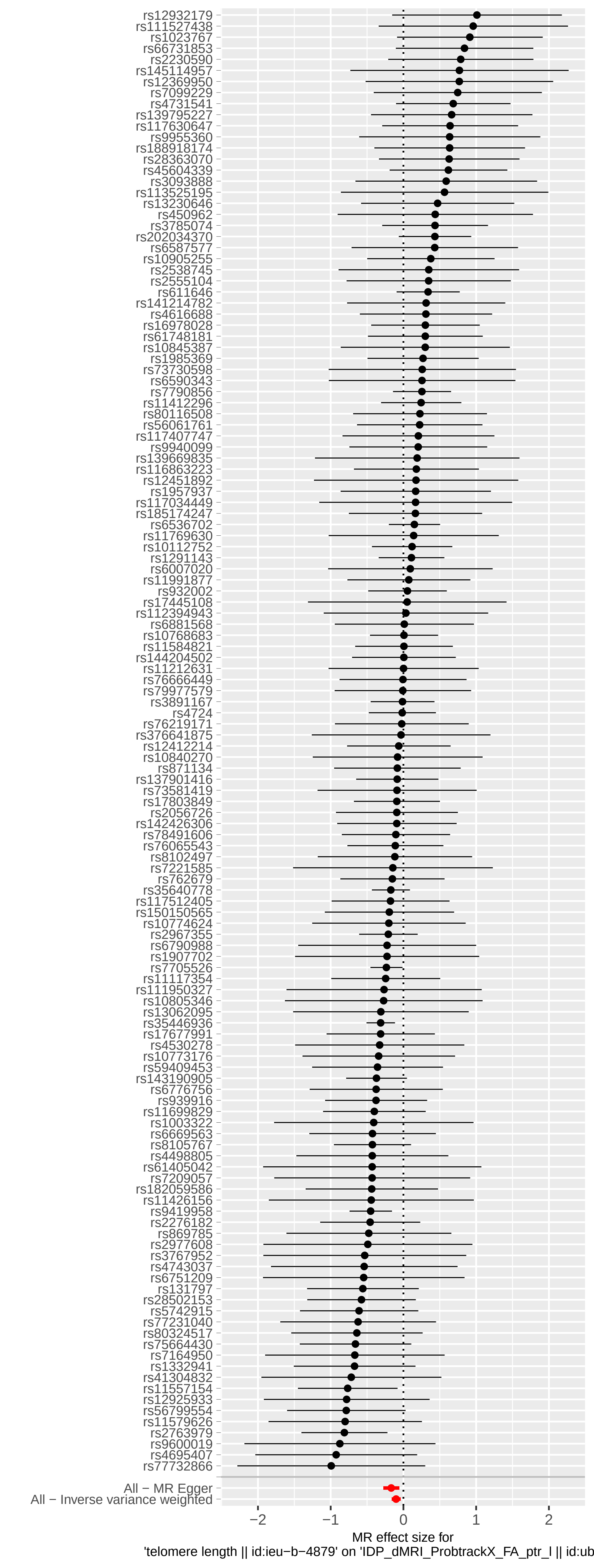
rs12932179
rs111527438
rs1023767
rs66731853
rs2230590
rs145114957
rs12369950
rs7099229
rs4731541
rs139795227
rs117630647
rs9955360
rs188918174
rs28363070
rs45604339
rs3093888
rs113525195
rs13230646
rs450962
rs3785074
rs202034370
rs6587577
rs10905255
rs2538745
rs2555104
rs611646
rs141214782
rs4616688
rs16978028
rs61748181
rs10845387
rs1985369
rs73730598
rs6590343
rs7790856
rs11412296
rs80116508
rs56061761
rs117407747
rs9940099
rs139669835
rs116863223
rs12451892
rs1957937
rs117034449
rs185174247
rs6536702
rs11769630
rs10112752
rs1291143
rs6007020
rs11991877
rs932002
rs17445108
rs112394943
rs6881568
rs10768683
rs11584821
rs144204502
rs11212631
rs76666449
rs79977579
rs3891167
rs4724
rs76219171
rs376641875
rs12412214
rs10840270
rs871134
rs137901416
rs73581419
rs17803849
rs2056726
rs142426306
rs78491606
rs76065543
rs8102497
rs7221585
rs762679
rs35640778
rs117512405
rs150150565
rs10774624
rs2967355
rs6790988
rs1907702
rs7705526
rs11117354
rs111950327
rs10805346
rs13062095
rs35446936
rs17677991
rs4530278
rs10773176
rs59409453
rs143190905
rs6776756
rs939916
rs11699829
rs1003322
rs6669563
rs8105767
rs4498805
rs61405042
rs7209057
rs182059586
rs11426156
rs9419958
rs2276182
rs869785
rs2977608
rs3767952
rs4743037
rs6751209
rs131797
rs28502153
rs5742915
rs77231040
rs80324517
rs75664430
rs7164950
rs1332941
rs41304832
rs11557154
rs12925933
rs56799554
rs11579626
rs2763979
rs9600019
rs4695407
rs77732866
All − MR Egger
All − Inverse variance weighted
−2
−1
0
1
2
MR effect size for
'telomere length || id:ieu−b−4879' on 'IDP_dMRI_ProbtrackX_FA_ptr_l || id:ub
